# Atomic-scale structure of interfacial water on gel and liquid phase lipid membranes†

**DOI:** 10.1039/d3fd00094j

**Published:** 2023-06-19

**Authors:** Simone Benaglia, Harriet Read, Laura Fumagalli

**Affiliations:** a Department of Physics and Astronomy, University of Manchester Manchester M13 9PL UK simone.benaglia@manchester.ac.uk; b National Graphene Institute, University of Manchester M13 9PL UK

## Abstract

Hydration of biological membranes is essential to a wide range of biological processes. In particular, it is intrinsically linked to lipid thermodynamic properties, which in turn influence key cell functions such as ion permeation and protein mobility. Experimental and theoretical studies of the surface of biomembranes have revealed the presence of an interfacial repulsive force, which has been linked to hydration or steric effects. Here, we directly characterise the atomic-scale structure of water near supported lipid membranes of 1,2-dimyristoyl-*sn-glycero*-3-phosphocholine in their gel and liquid phase through three-dimensional atomic force microscopy (3D AFM). First, we demonstrate the ability to probe the morphology of interfacial water of lipid bilayers in both phases with sub-molecular resolution by using ultrasharp tips. We then visualise the molecular arrangement of water at the lipid surface at different temperatures. Our experiments reveal that water is organised in multiple hydration layers on both the solid-ordered and liquid-disordered lipid phases. Furthermore, we observe a monotonic repulsive force, which becomes relevant only in the liquid phase. These results offer new insights into the water structuring near soft biological surfaces, and demonstrate the importance of investigating it with vertical and lateral sub-molecular resolution.

## Abstract 2

The molecular arrangement of interfacial water upon solid-to-liquid phase transition of lipid molecules was investigated with sub-molecular resolution by 3D AFM.

## Graphical abstract

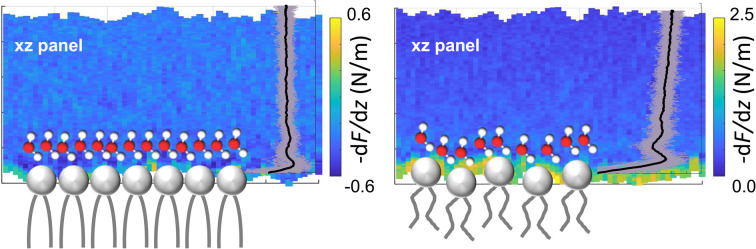

## 1 Introduction

The interaction of biological molecules with water is of paramount importance to their structure and function.^1,2^ Together with long-range electrostatic and van der Waals interactions, hydration forces are considered to be interfacial forces that are critical to biological systems, although their governing mechanism is still under debate.^3,4^ Particularly relevant and long studied is their role in the structure and function of biological membranes. The main constituents of biomembranes are lipids, which are amphiphilic molecules composed of hydrophobic alkyl tails and a hydrophilic head. When in water, they are thermodynamically driven to form bilayers with the hydrophilic heads exposed to the water. Importantly, the hydration structure and dynamics is known to determine the intermolecular and intermembrane interactions, whilst also contributing to the adaptation of their functions to new requirements. For instance, it is well known that different levels of hydration change the thermodynamics of lipid bilayers, *e.g.* their transition temperature, enthalpy, and entropy.^5^ These properties, in turn, regulate important physical processes occurring at the membranes, such as their permeability, lipid and protein mobility, signal propagation, phenomena of endocytosis and exocytosis, intermembrane adhesion and fusion, and adhesion on solid surfaces.^6–10^ Among these properties is the ability of lipid molecules to undergo phase transitions and generate domains within the membrane selective for specific biomolecules which play a key role in bioprocesses, such as the formation of lipid rafts.^11,12^ Below and above the main phase transition temperature, *T*_m_, van der Waals forces between hydrocarbon chains decrease and lipid molecules convert from a solid (gel) ordered *S*_o_ state, characterised by extended hydrocarbon tails and regular ordered packing within the bilayer, to a liquid (fluid) disordered *L*_d_ state, where the hydrocarbon chains compress and the lipids are more free to diffuse laterally and thus are not regularly packed within the bilayer. Moreover, this induced disorder of the hydrocarbon tails in the *L*_d_ state determines a morphological change with an increase of the membrane area alongside a decrease of its thickness.^13^ Whilst structural characterisation has provided important information on lipid phase transitions,^14^ their hydration properties have remained elusive due to the inherent experimental difficulties in probing them.

Hydration of biological membranes has been studied by different technical approaches, such as osmotic stress experiments, which are able to quantify the pressure between lipid membranes in multilamellar vesicles,^15^ and the surface force apparatus (SFA), measuring force *vs.* distance curves between two opposed lipid bilayers or between a solid and bilayer surfaces.^8,16^ Experiments performed with both techniques consistently revealed the presence of an exponential repulsive force at the lipid surface due to a combination of hydration and steric forces. Moreover, this tends to increase for lipids in their *L*_d_ phase. Similar conclusions have also been obtained from molecular dynamics (MD) simulations.^17,18^ Importantly, spectroscopic studies have led to relevant observations of hydration at membranes, such as the direct quantification of the number of water molecules per lipid, together with their dynamics and orientation.^19–21^ Recently, a deeper analysis of the interplay between hydration and lipid phase transition has been carried out experimentally by non-resonant angle-resolved second harmonic scattering,^22^ and differential scanning calorimetry,^23^ and theoretically by MD simulations.^18,24^ In particular, Garcia *et al.* have provided evidence that the main phase transition of phosphatidylcholine (PC) membranes is associated with a partial disruption of the water hydrogen bonds formed at the lipid surface.^23^ Experimentally, these techniques suffer a major limitation, that is, they can only study the behaviour of the membrane on the large scale, thus averaging out the contribution of single molecules, and cannot visualise the organisation of water molecules at the lipid surface. Moreover, the roughness of the lipid surface due to corrugation of the lipid headgroups and thermal fluctuations cannot be taken into account. This is why, despite many scientific studies on biomembrane hydration, little is known on the atomic-scale structure of the water–lipid interface.

**Fig. 1:**
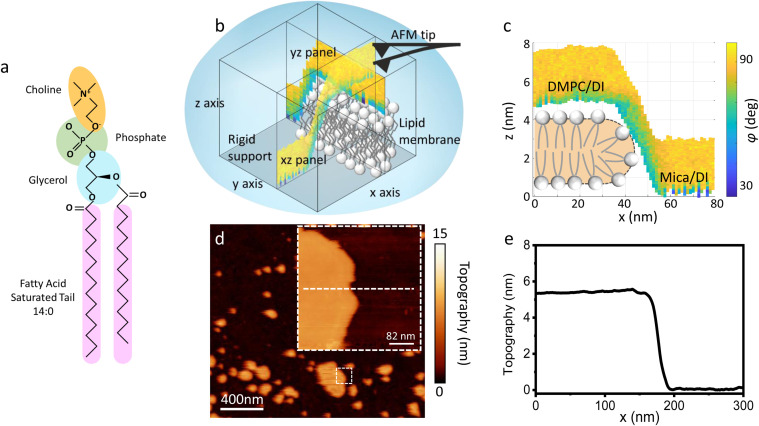
(a) Molecular structure of a DMPC lipid. (b) Schematic of the 3D AFM imaging process. The AFM cantilever, oscillated with a photothermal excitation, moves laterally in an *xy* raster scan with the addition of a sinusoidal *z*-modulation. The picture shows an *xz* and *yz* panel, orthogonal to each other, which are part of the same data cube obtained over a DMPC lipid bilayer patch. (c) Visualisation of the *xz* panel shown in (b), showing the phase contrast during 3D AFM imaging. The height of the DMPC bilayer can be reconstructed. A cartoon depicting the organisation of lipids in SLBs is included.^25^ (d) Standard AM AFM topographic image obtained on the same patch mapped with 3D AFM in (b and c). The inset is a zoomed-in area of the larger image. (e) Height profile across the white dashed line in (d). The lipid bilayer height obtained in standard AM AFM nicely matches the one derived from the 3D AFM data.

To address this problem, here we used three-dimensional atomic force microscopy (3D AFM), a powerful technique that allows probing of the solid–liquid interfacial region with 3D (*i.e.* vertical and lateral) atomic scale resolution.^26^ Similarly to SFA experiments, this is achieved by recording changes in the force sensed by the probe due to density variation of the liquid molecules at the sample surface. However, here, we made use of ultrasharp tips, enabling us to gain information of the water interface variation in all three positional directions, as well illustrated in Fig. 1. Whilst in recent years 3D AFM has gained much popularity to map the solid–liquid interface of flat, stiff, crystalline surfaces, with examples spanning from mica, calcite, gibbsite and other van der Waals materials,^26–31^ only recently has it been applied to the study of soft biological molecules. The latter is much more challenging due to the soft nature of the interface. This is because as the AFM tip indents such soft surfaces, the sample hydration layers can be disrupted. Despite this, it has already shown great potential in mapping the water interface of globular and membrane proteins, DNA, and lipids.^32–36^ In particular, the work of Fukuma and colleagues,^35,36^ revealing for the first time the 3D water structure at lipid bilayer surfaces, is very relevant for our study. Building on these studies, here we experimentally investigate the structural arrangement of water molecules at the interface of the zwitterionic lipid 1,2-dimyristoyl-*sn-glycero*-3-phosphocholine (DMPC, Fig. 1a), above and below the main phase transition. First, we showed that by carefully tuning the AFM parameters, we were able to probe the water–lipid interface without perturbing it. Subsequently, we carried out temperature controlled experiments, above and below *T*_m_, where the water arrangement over the lipid bilayer in both their *L*_d_ and *S*_o_ phase was mapped, comparing their hydration structures with 3D atomic scale resolution. We show that although the interface organises into multiple hydration layers near the soft hydrophilic lipid heads on both phases, it is affected by the thermodynamic phase of the lipids, with a monotonic repulsive trend characterising the force probed on the *L*_d_ phase which is minimised below the *T*_m_. Notably we demonstrate, for the first time, the presence of oscillatory hydration layers at the interface of *L*_d_ phase lipid membranes, which have remained elusive in previous attempts.^36^ A discussion of the mechanism underlying the observed phenomena follows. The results shown here provide new experimental data of the interfacial water structure at the lipid membranes, with relevant implications for cell processes occurring at the interface.

## 2 Materials and Methods

### 2.1 Sample preparation

Supported lipid bilayers (SLBs) of DMPC were prepared for AFM measurements *via* vesicle fusion techniques described in previous literature.^37^ First, dried lipid powder (Avanti Polar Lipids Inc, USA), stored away from light at −20 °C, was dissolved in chloroform (anhydrous, 99%, Sigma-Aldrich) to a stock solution of concentration ≈5 mg ml^−1^. The lipid-chloroform solution was then evaporated under a stream of nitrogen, forming a lipid-film on the walls of the glass vial before re-hydrating with deionised (DI) water of resistivity 18.2 MΩ (Millipore) and forming multi-lamellar vesicles (MLVs). Stock solutions were subsequently sonicated to form solutions of uniformly sized MLVs, from which the desired concentrations for deposition were made. For 3D AFM measurements, SLBs were formed on hydrophilic substrates of mica and silicon oxide. Mica surfaces were freshly cleaved before use. For the silicon wafers, a piranha solution of 90% sulphuric acid (99.9% Sigma-Aldrich), and 10% hydrogen peroxide (Sigma-Aldrich) at 80 °C was used as a cleaning protocol, whereby the wafers were added to the solution for a maximum of two minutes before removing and rinsing with DI water thoroughly. Prior to the vesicle deposition, we sonicated the silicon chips with a solution (5% in DI water) of Decon-90 (Decon Laboratories Ltd, UK) to remove any additional residues from the surface. For the formation of SLBs, 100 μL of diluted liposome/DI solutions, of approximately 0.2 mg ml^−1^, were deposited upon the substrates and left to incubate for ten minutes at room temperature. Following this, the surface was thoroughly rinsed with DI water and if necessary, was exchanged for an imaging solution of 100 mM KCl.

### 2.2 AFM measurements

Amplitude modulation (AM) AFM was used for both 2D and 3D AFM measurements on DMPC SLBs in DI water, carried out using a commercial AFM (Cypher ES, Asylum Research, Oxford Instruments, UK). HQ NSC19/Cr–Au (Mikromasch, Bulgaria) cantilevers were selected for standard 2D AM AFM imaging whilst Arrow UHFAuD (NanoWorld, Switzerland) cantilevers were used for 3D AFM. Both cantilever types were driven with a photothermal excitation in their first eigenmode. We calibrated the AFM cantilever for 3D AFM measurements using the Sader’s method^38^ implemented within the commercial software of our AFM (“GetReal”). Calibrated values in DI water of the spring constant (*k*), resonance frequency (*f*), and quality factor (*Q*) were in the range *k* = 9.4–17.3 N m^−1^, *f* = 555–779 kHz, *Q* = 6. Small amplitude oscillations (approximately 100–500 pm) were typically chosen for 3D AFM operation.

#### 2.2.1 AFM characterisation of SLBs *T*_m_

In order to determine the *T*_m_ of the SLB between its *S*_o_ and *L*_d_ phase, temperature controlled 2D AM AFM measurements were performed. The temperature was cycled between 15 °C and 36 °C at a heating rate of approximately 0.5 °C s^−1^, allowing for the sample to equilibrate for approximately 5 minutes before performing AFM topography measurements. Furthermore, consecutive AFM images were taken until the sample was at equilibrium. To find the transition temperature, the Van’t Hoff equation, which is used widely to describe thermodynamic systems, was employed to fit the data, as described in previous literature.^7^ Briefly, for a transition between two states, an equilibrium constant, *K*, may be defined such that *K* = *s*/*l* where *s* and *l* are the fractional occupancies in the *S*_o_ and *L*_d_ states, respectively. A modified version of the van’t Hoff equation expressed in terms of *s* may be then written as:^39,40^1
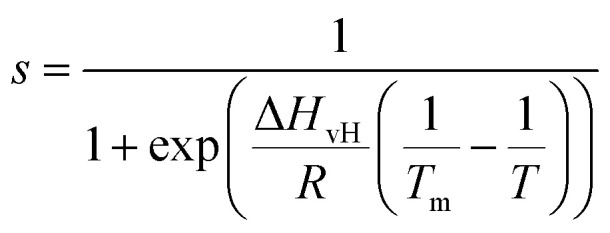
where *T* is the temperature, Δ*H*_vH_ is the van’t Hoff enthalpy of the transition, and *R* is the universal gas constant. Thus, by determining the fractional occupancy of the lipids in their *S*_o_ phase at different temperatures, and fitting a sigmoidal curve (eqn (1)), we extracted the transition temperature of the SLB.

#### 2.2.2 3D AFM measurements

We performed 3D AFM using the AC Fast Force Map (FFM) mode available within the AFM software (Asylum Research, Oxford Instruments, UK), whereby the phase and amplitude of the cantilever oscillations over a pre-determined *xy* grid at different *z* values were recorded, thus yielding a 3D data cube. Typically, small scans (1–5 nm) were performed with 64 × 32 pixels in the *xy* plane, and 2000 pixels in the *z*-direction. The *z*-modulation was typically between 2–5 nm in size, with a frequency of 250 Hz, achieving a 3D data set within approximately 20 seconds. 3D AFM measurements were performed at a temperature of 15 °C and 25 °C for lipid membranes in their *S*_o_ and *L*_d_ phase, respectively (see below for discussion regarding the phase transition). We developed fully-customised Matlab software to process the raw data, which allows the 3D visualisation of the data, the extraction of each *xz* or *yz* panel of the 3D data cube and of single curves, and calculation of average curves, as shown below. We presented the obtained 3D AFM results in terms of the interaction stiffness, or force gradient, of the tip-sample interaction. From the recorded AFM observables, the gradient of the force, −d*F*/d*z*, between the tip and the lipid–liquid interface may be reconstructed as defined in literature (see also ESI†).^41,42^

## 3 Results and discussion

### 3.1 3D AFM on lipid bilayers

As previously mentioned, 3D AFM allows for a volumetric reconstruction of the interface between the aqueous solution and the lipid bilayer. A representative 3D data cube obtained on a SLB formed on mica is shown in Fig. 1b, where two orthogonal *xz* and *yz* panels, *i.e.* panels perpendicular to the sample surface, are plotted. These panels contain the information about the interface formed between the sample and the water above. The *xz* panel is better visualised in the 2D image in Fig. 1c. Here, a phase panel allows the reconstruction of the topographic features of the lipid bilayers formed on mica. The SLB of mica as well as the interface on the SLB are reconstructed. It can be seen that throughout the scans, the SLB has a thickness of ≈5 nm. This is consistent with the height obtained with 2D AFM imaging prior to and after performing 3D AFM measurements, an example of which is shown in Fig. 1d and e, and with the expected thickness for DMPC bilayers.^13^ The cross section highlights good agreement between the height obtained with 3D AFM and the 2D AFM scans. Hence, we deduce that the SLB has not been deformed or damaged during the measurement. Although the parameters have been optimised for such a large scan (≈80/100 nm) to properly track the topographic features of the SLBs, it is quite difficult to extract detailed information of the interface between the SLB and water on this lateral scale, as seen in Fig. 1c. Hence, we performed measurements over smaller areas of the SLB (<5 nm), which allowed us to obtain details of the interfacial water at higher resolution.

### 3.2 Interfacial water and molecular resolution on lipid bilayers

**Fig. 2:**
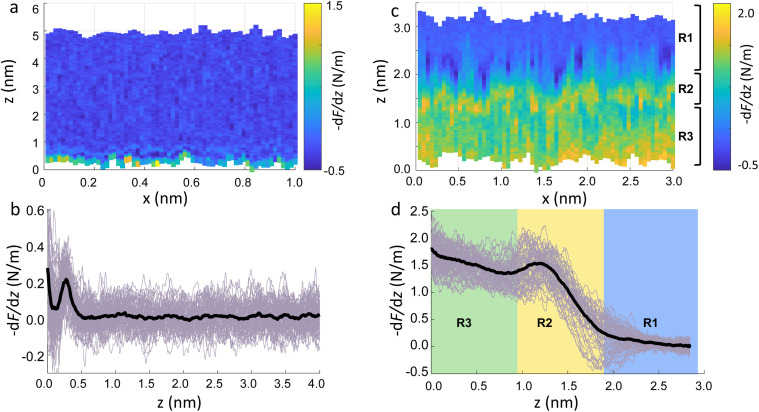
(a) Force gradient *xz* panel obtained at the interface of DMPC SLBs in the *S*_o_ phase in DI water. (b) Corresponding force gradient *vs.* tip-sample distance profile of the panel in (a). The average curve is plotted as a black thick line. (c) *xz* panel of the SLBs/DI water interface, obtained upon increasing the force applied to the sample, causing its deformation. (d) Force gradient *vs.* tip-sample distance profile relative to the panel in (c). In (c and d) R1 is the liquid interface, R2 represents the region where the tip indents into the lipid heads, R3 corresponds to the indentation of the alkyl chains.

Fig. 2a show an *xz* panel representing the tip-sample interaction (force gradient) for a DMPC bilayer in DI water, together with its corresponding one dimensional (1D) plot (Fig. 2b), recorded at 15 °C. At this temperature, the SLBs were considered to be in their *S*_o_ phase (see below for further discussion regarding the *T*_m_ of DMPC SLBs). The *xz* panel shows a stripe extending laterally throughout the panel above the lipid surface. This is interpreted with the presence of one hydration layer within the first 0.5 nm closest to the surface. This can be further visualised in the 1D plot (Fig. 2b). Here, the stripe seen in the *xz* panel corresponds to a maximum in the force gradient.

In the results shown, the force gradient follows a similar trend to the one previously observed in 3D AFM measurements of the interface of crystalline materials and water or electrolyte solutions, that is, composed of a superposition of an oscillatory and a monotonically decaying force.^43^ The oscillatory trend can be interpreted following the commonly accepted solvent tip approximation (STA) model.^44^ Alternating regions of attractive and repulsive regimes up until a contact point, correlate with the interaction between the hydration layer on the hydrophilic tip and the structured hydration layers on the substrate surface. Hence, we argue that the maximum here in the force gradient is linked to a maximum in the water density profile, as previously shown for other PC lipids in their *S*_o_ phase.^35,36^ The region of the force profile where no interaction forces are detected is considered as the interaction of the hydration around the tip with the bulk water (where no high local density of water is expected). Additional measurements proved consistently the presence of not only a single, but multiple hydration layers (see below and ESI, Fig. S3†). The monotonically decaying trend of the force will be further discussed below. Note that, despite the similarity with respect to previous measurements obtained on crystalline materials, our experiments are performed under different (and challenging) conditions, as the surface of the lipid membrane is much rougher and the lipid headgroups are prone to thermal fluctuations. Moreover, due to the soft nature of the sample, there arises a potential problem of penetrating inside the lipid bilayer itself during 3D AFM spectroscopy.

To demonstrate that the features measured in 3D AFM are indeed hydration layers over the lipid heads, and not a cantilever response to the possible deformation of the SLB, we performed experiments applying higher forces. Fig. 2c shows an *xz* panel with three distinct regions recognised as R1, R2, and R3. The first area (R1) at larger distances (from ≈1.8 nm to ≈3 nm), corresponds to the interfacial water region. The second (R2, from ≈1 nm to ≈1.8 nm) reveals the presence of a more intense stripe (bright yellow color) followed by the third region (R3, from 0 nm to ≈1 nm) characterised by a continuous increase of the force. The high intensity stripe seen in the *xz* panel corresponds to a maximum in the force gradient plot (Fig. 2d) with a thickness of ≈0.8 nm. This is close to the value expected for the steric headgroup thickness of DMPC.^13^ Hence, we concluded that R2 corresponds to full penetration into the lipid heads, whilst R3 corresponds to the indentation of the tip into the remaining part of the membrane, *i.e.* within the lipid tails.

Our experiments show that by increasing the applied force, it was possible to directly indent into the membrane and distinguish between the indentation of the lipid headgroups and of the lipid tails. Asakawa *et al.* previously performed similar experiments on a DPPC interface.^35^ They visualised multiple oscillation layers at the interface with the lipid membrane and attributed the first oscillatory peak closest to the surface to the deformation of the lipid headgroups. Our data nicely match their results and highlight the ability of 3D AFM to perform morphological studies of biological specimens with molecular resolution.

### 3.3 Interfacial water structure on lipid bilayers in *S*_o_ and *L*_d_ phase

**Fig. 3:**
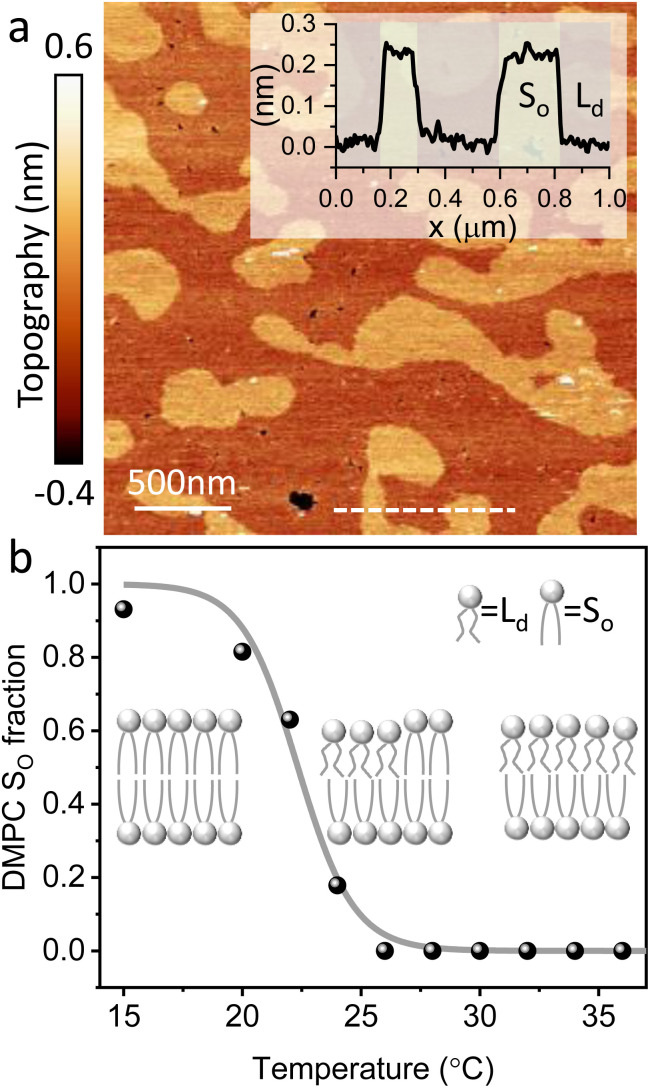
(a) Topography image of DMPC SLBs obtained on mica at 22 °C. The map shows regions with different heights as highlighted in the cross section (inset) obtained along the dashed line. The taller regions consist of lipids in their *S*_o_ phase, whilst in the smaller regions the distal leaflet has undergone its phase transition to the *L*_d_ phase. (b) By recording the fractional area occupied by the *S*_o_ phase *vs.* the temperature, it is possible to extract the *T*_m_. The fitting with the Van’t Hoff equation is shown as a grey line. A schematic representing the transition of the distal leaflet is provided.

The main phase transition of lipid bilayers is characterised by the loss in lateral order due to the melting of the lipid alkyl tails. This is followed by an increase in the bilayer area and decrease in the bilayer thickness, which can be monitored by AFM.^39,40^ An example of that is shown in Fig. 3a. Here, we display the topography map of the lipid bilayer during the phase transition. Regions of different height are linked with the simultaneous presence of the *S*_o_ and *L*_d_ phase, being the domains of larger and smaller thickness in the *S*_o_ and *L*_d_ phase, respectively. This is further highlighted by the cross section taken along the white line in the topography map. The difference in height between the lipids in their *S*_o_ and *L*_d_ phase is ≈0.3 nm. This value points to a transition of only the lipid distal leaflet, as previously discussed in the literature.^40,45^ We monitored the transition of the distal leaflet with AFM by changing the temperature in steps between 15 °C and 36 °C and leaving the sample to equilibrate at each temperature before commencing with the next AFM image. Hence, we proceeded by quantitatively evaluating the area occupied by the *S*_o_ and *L*_d_ phase and calculating the fraction occupied by the two phases (as explained in the Materials and methods section). Fig. 3b shows the plot of the DMPC *S*_o_ fraction *vs.* temperature. The data can be evaluated with a modified version of the Van’t Hoff equation^39,40^ to determine the *T*_m_. The fitting is shown in grey, and the value extracted for *T*_m_ is 22.2 ± 0.1 °C, as expected for DMPC lipids.^39,46^ Once the *T*_m_ was precisely determined we could proceed with 3D AFM measurements on SLBs where the distal layer was either entirely in its *S*_o_ or *L*_d_ phase, with the specific purpose to characterise differences in their hydration.

**Fig. 4:**
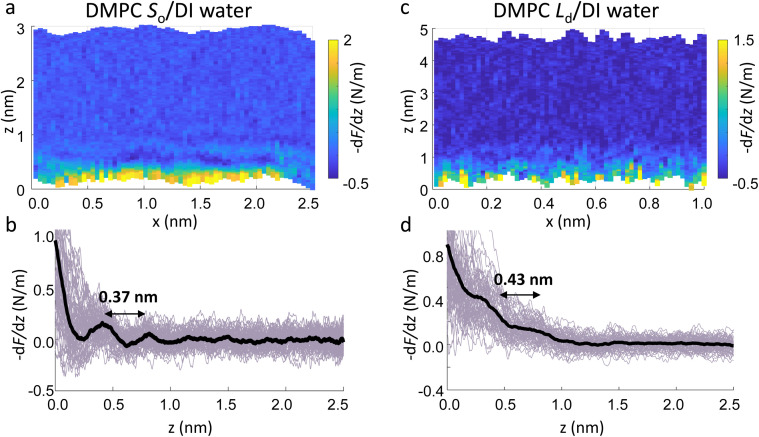
Force gradient *xz* panels obtained at the interface of DMPC SLBs in the *S*_o_ phase (a) and *L*_d_ phase (c) in DI water. The corresponding force *vs.* tip-sample distance profiles are shown in (b) and (d), respectively. The average curve is plotted as a black thick line. Both interfaces are characterised by two hydration layers within the first nm from the lipid surface.

Fig. 4a and c show two representative *xz* force gradient panels obtained on the *S*_o_ and *L*_d_ phases of DMPC bilayers, with their corresponding 1D plots (Fig. 4b and d), respectively. Both 3D AFM *xz* panels show the typical features corresponding to hydration layers. Indeed, from the plot it is possible to notice the presence of two hydration layers in both the *S*_o_ and *L*_d_ phase, with interlayer distances of 0.37 nm and 0.43 nm, respectively. Experiments were performed with the same tip on the two phases to minimise the impact of different tip radii, and with different tips on multiple samples to ensure reproducibility of the data. Considering all of the experimental measurements performed over the two lipid phases, we observed the presence of one or more hydration layers in addition to sets of data where no hydration structures were visualised. Amongst the maps with clear hydration layers, we calculated the occurrence frequency of one or two hydration layers being 54% and 46% for the *S*_o_ phase and 77% and 23% for the *L*_d_ phase, revealing that two hydration layers were more easily detected on the *S*_o_ than on the *L*_d_ phase. When two hydration layers occurred, the distance between the layers was on average 0.40 ± 0.03 nm and 0.44 ± 0.03 nm, for the *S*_o_ and *L*_d_ phase (see also the box plot in ESI, Fig. S6†). Importantly, smaller interlayer distances (≈0.35 nm, as the one shown in Fig. 4a) could be found only on the *S*_o_ phase, whilst the *L*_d_ interfacial layers were characterised by larger interlayer distances. This value is comparable to that previously found with a similar AFM technique for the *S*_o_ phase of DPPC bilayers in aqueous solution,^35^ although larger than expected for the diameter of the water molecule (≈0.3 nm). Despite larger interfacial hydration layers previously found on hydrophobic materials,^30,34,47^ here DMPC lipids are zwitterionic, with heads that have a hydrophilic character. Hence, we attribute the larger interlayer distance to the intrinsic roughness and thermal motion of the lipid molecules within the bilayer. Indeed, if the lipid–water interface were flat and smooth, the density (and force) profile would exhibit oscillations close to the packing of planar water layers on top of the surface. This is observed, for example, on hydrophilic and atomically flat crystalline materials, such as mica, where periodicities closer in size to the water molecule diameter are seen, which are linked to a stronger network of hydrogen-bonds between water molecules in the same ordered layer.^26^ Instead, SLBs have a roughness comparable to the scale of the water molecules, which is likely to disrupt the hydrogen-bond network formed by the water molecules and reduce the structuring within the hydration layers. This determines the strong suppression of the oscillatory force as mapped in force-based techniques as AFM spectroscopy or SFA. Similar conclusions have been deduced for the interfacial water structures formed at amorphous surfaces such as silicon.^29^ For the difference between the two phases, we speculate that smaller interlayer distances and multiple hydration layers were more easily visualised on the *S*_o_ phase, probably due to a reduced thermal motion of the lipids, which causes a more solid-like material with an increased order and higher breakthrough force – the highest force that the bilayer can withstand before breaking due to the tip penetration.^48^ Again, as for the case of crystalline materials, an increased order of the probed surface facilitates the visualisation of hydration structures in 3D AFM.

**Fig. 5:**
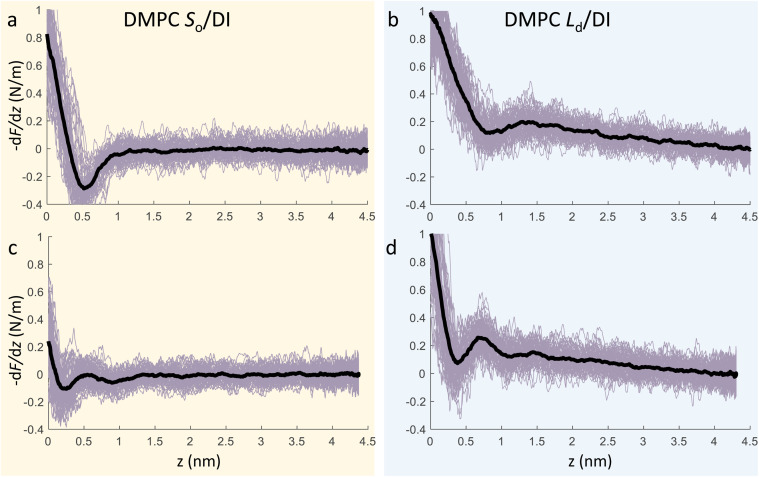
3D AFM force gradient *vs.* tip-sample distance profiles obtained at the interface of a DMPC SLB in its *S*_o_ phase (a, c) and *L*_d_ phase (b, d). During 3D AFM imaging, some panels did not show any oscillatory hydration structures but only the monotonic contribution to the force (a, b), whilst in others the presence of hydration layers was evident (c, d). The average curve is plotted as a black thick line.

We now focus on the specific behaviour of the force obtained on the *S*_o_ and *L*_d_ phases. We analysed the experimental data in depth to characterise not only the typical interlayer distance of the hydration layers, but also to evaluate the monotonic decay of the force on the two different phases. Fig. 5 shows four additional 1D panels obtained on lipids in the *S*_o_ and in the *L*_d_ phase. As already mentioned, we found areas where only a monotonic force was probed, as in Fig. 5a and b, and other areas where the force had the two aforementioned contributions (monotonic + oscillatory), as depicted in Fig. 5c and d. The *xz* panels corresponding to the 1D plots of Fig. 5 are shown in the ESI, Fig. S2.† To better quantify the monotonic contribution to the force, we fitted the data to an empirical function which combines the monotonic exponential decay *λ*_m_ with the oscillatory contribution,^3,43,49,50^ as *F*(*z*) = *F*_o_ cos(2π*z*/*d* + *ϕ*)e^−*z*/*λ*_o_^ + *F*_m_e^−*z*/*λ*_m_^. Here, *λ* and *d* are the decay length and liquid molecular diameter, with o and m subscripts qualifying the oscillatory and monotonic contribution, respectively. The decay length for the *S*_o_ phase turns out to be *λ*_m,*S*_o__ = 0.28 ± 0.12 nm, and for the *L*_d_ phase *λ*_m,*L*_d__ = 0.64 ± 0.49 nm (the distribution of *λ*_m_ for the two phases together with an example of the fitting procedure is shown in the ESI, Fig. S5 and S6†). Our data clearly show that independently of the presence of hydration layers, an extended monotonic repulsive force is ubiquitous when the water interface of *L*_d_ phase lipids was probed, which decreased at the water interface of lipids in their *S*_o_ phase. Even though a repulsive monotonic background might originate from DLVO forces (electrostatic + van der Waals), this would not explain its absence upon phase transition. Moreover, additional experiments performed in DI water, and consequently in 100 mM KCl, did not see any modification of the trend of the force gradient curve (Fig. S4†). This independent behaviour excludes the force probed in 3D AFM with very sharp tips, as in our case, to be of electrostatic/van der Waals origin (the Debye length would decrease 100 times), and confirms previous observations obtained with similar AFM tips on crystalline materials for electrolyte concentrations below saturation.^51,52^ The repulsive monotonic force contribution probed on the interfacial water of the *L*_d_ phase might be better explained by considering a combination of relevant phenomena that occur upon phase transition: (i) the increased thermal thickness fluctuations of the lipid bilayers;^16,53^ and (ii) the softening of the bilayer in the *L*_d_ phase.^48^ It is difficult to determine which of the two is the dominant effect, nor can we exclude a combination of additional temperature dependent physical factors.^53^ Importantly, although it has been proved a greater affinity of the *L*_d_ phase lipid molecules to water and hence a higher hydration of lipids,^54,55^ we exclude a connection to the increased monotonic trend of the force in our measurements. Indeed, an increased repulsion would signify a greater order of the water molecules upon raising the temperature, which is unlikely to be happening. Similar conclusions have been drawn for complementary experiments obtained with the SFA and AFM where short-range repulsive forces above *T*_m_ were mainly considered to be induced by steric repulsion attributed to thermal motions of head groups and thickness fluctuations of the *L*_d_ bilayer.^16,36^ It is important to note that none of the previous studies have demonstrated the presence of clear organised interfacial water layers on *L*_d_ lipid bilayers, as observed here. We achieved that with the use of cantilevers with very sharp tips and with higher sensitivity, oscillated at small amplitudes, which were crucial to such results.

**Fig. 6:**
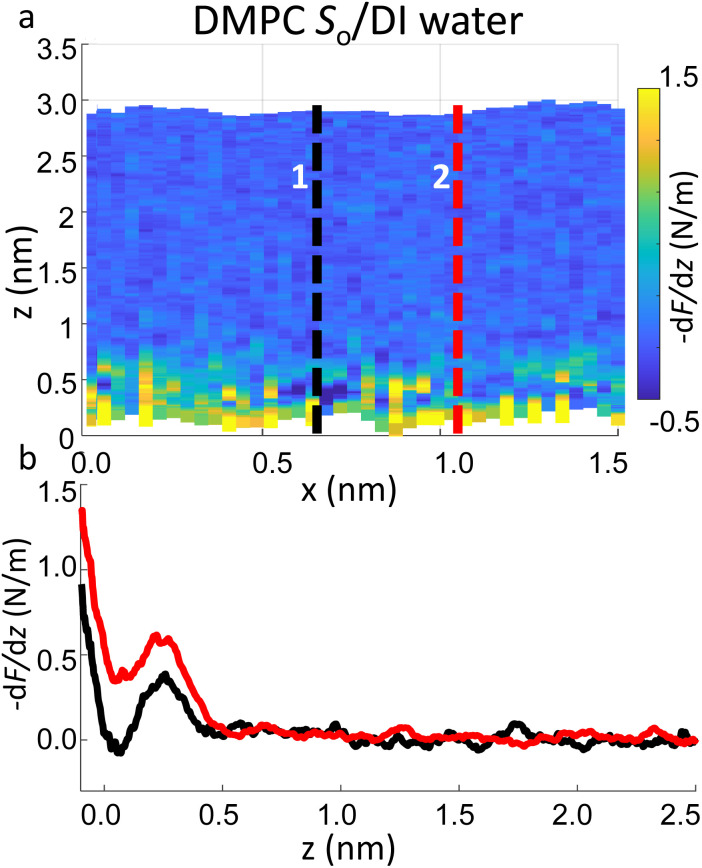
(a) 3D AFM force gradient *xz* panel showing lateral resolution of the interfacial hydration at the surface of *S*_o_ lipids. Force gradient *vs.* tip-sample distance profiles obtained at position 1 and 2 in (a) are plotted in (b). Whilst in profile 2 (red curve) a hydration layer and monotonic repulsion are both evident, only the latter contribution disappears in profile 1 (black curve).

Importantly the data obtained on the *S*_o_ phase in some cases show the complete absence of the monotonic background force seen for the water interface of the *L*_d_ phase. This may seem in contradiction with the data previously shown on the same phase (Fig. 2a and ESI Fig. S3†), where a repulsive monotonic trend of the force was observed within the first 0.5 nm from the surface. This can be better understood by considering the data in Fig. 6. As previously mentioned, it is expected that the alternation of lipid heads determines a significant topographical roughness of the water interface of lipid molecules, which in turn causes a lateral change of the hydration structure probed at the interface. Fig. 6 displays two force gradient curves taken on the same panel at two different *x* positions (along the dashed lines). Although the two curves show similar oscillatory behaviour, the monotonic repulsive trend tends to disappear at position 1 (black curve) with respect to position 2 (red curve). To our knowledge, these are the first measurements showing local differences of the monotonic hydration contribution at the lipids–water interface. This observation may explain the observed reduction in the monotonic exponential force found here as well as in osmotic pressure and SFA experiments,^8,16,56^ which laterally average out the contribution of the water over the full membrane. This underlines the importance of evaluating spectroscopic data not only in the vertical but also in the lateral direction with atomic-scale resolution, which is one of the main advantages of 3D AFM.

## 4 Conclusion

In summary, we demonstrated that carrying out 3D AFM using ultrasharp tips is an effective tool to visualise the interfacial water structure formed at lipid bilayer interfaces in its solid and liquid phases. We showed that, despite the soft nature of the lipid, it is possible to limit the deformation of the bilayer in both its *S*_o_ phase and *L*_d_ phase whilst performing 3D AFM experiments using small excitation amplitudes of the cantilever, and thus applying low forces to the sample. We demonstrated that by adjusting the force applied to the lipid bilayer, 3D AFM measurements can be fine tuned to allow for a direct observation of the nanometric morphological features of the lipids (heads and tails) in addition to the layered structures formed by the interfacial water molecules. Moreover, our AFM measurements proved that the interaction of a nanometric tip with the lipid surface is, in general, characterised by an oscillatory component and an exponential monotonic decay, in agreement with previous force-based measurements obtained on lipid membranes. Whilst the former can be easily linked to the position of high density water layers in the vicinity of the lipid surface, the latter cannot be so easily explained by only considering the presence of hydration. Importantly, although we visualised 1–2 hydration layers at the surface of both lipids in their *S*_o_ phase and *L*_d_ phase, multiple water layers were more easily found on bilayers in their *S*_o_ phase. By quantitatively evaluating the trend of the tip-sample force, a different behaviour was clearly observed for the two phases, as the force probed at the interface of *L*_d_ phase lipids showed a greater monotonic exponential decay with respect to that probed on the *S*_o_ phase. This observation matches previous literature reports, and can help to understand the origin of such repulsive force. The fact that this increases by raising the temperature of the system rules out that it originates from restructuring of the water itself. More likely, this is linked to effects that scale with temperature, such as thermal fluctuations of the lipid molecules themselves, as pointed out by Israelachvili and Wennerström,^53^ and softening of the bilayer. These observations show the importance of experimentally characterising interfacial forces on biomembranes (and possibly other biomolecules) with molecular scale resolution, as done in this study.

## Author contributions

S. B. and H. R. performed sample preparation, measurements and data analysis. L. F. supervised the project. S. B. wrote the paper with the help of H. R. and inputs of L. F.

## Conflicts of interest

There are no conflicts to declare.

## Supplementary Material

FD-249-D3FD00094J-s001
