# Supplementary material for: Atomic-scale structure of interfacial water on gel and liquid phase lipid membranes
Source: Faraday Discuss. 2023 Jun 19;249:453–68. doi: 10.1039/d3fd00094j (PMC10845012; doi:10.1039/d3fd00094j)
Supplement: FD-249-D3FD00094J-s001 [file FD-249-D3FD00094J-s001.pdf]

Electronic Supplementary Material (ESI) for Faraday Discussions.
This journal is © The Royal Society of Chemistry 2023

Electronic Supplementary Information

Atomic-Scale Structure of Interfacial Water on Gel and Liquid Phase Lipid Membranes

Simone Benaglia,[a,b*] Harriet Read,[a,b] and Laura Fumagalli[a,b]

[a] *Department of Physics and Astronomy, University of Manchester, Manchester, M13 9PL, UK;*[b] *National Graphene Institute, University of Manchester, M13 9PL, UK;* [*]*E-mail: simone.benaglia@manchester.ac.uk*

Contents

S1 Reconstruction of the force gradient from 3D AFM data **S2**

S2 3D AFM on S_o and L_d lipid phase **S3**

S3 Force independence of the salt concentration **S4**

S4 Fitting procedure for force gradient vs distance curves **S5**

S1 Reconstruction of the force gradient from 3D AFM data

In amplitude modulated 3D AFM the observables are the amplitude and the phase of oscillation of the cantilever. The interaction stiffness, K, or force gradient, $-dF/dz$, can be extracted as explained in the literature.[1,2] In particular, it is defined in terms of the AFM observables as:

$$K = -\frac{dF}{dz} = -k\left(1 - \left(\frac{f_d}{f}\right)^2\right) + \frac{kA_0}{AQ}cos(\phi) \tag{1}$$

where f and f_d are the driving and resonance frequencies of the cantilever (usually chosen to be $f = f_d$), k and Q are the spring constant and quality factor of the AFM cantilever, and A, A_0 and ϕ are the amplitude, free oscillation amplitude and phase shift. Figure S1 shows the amplitude and phase xy panel and the reconstructed force gradient of the lipid/water interface.

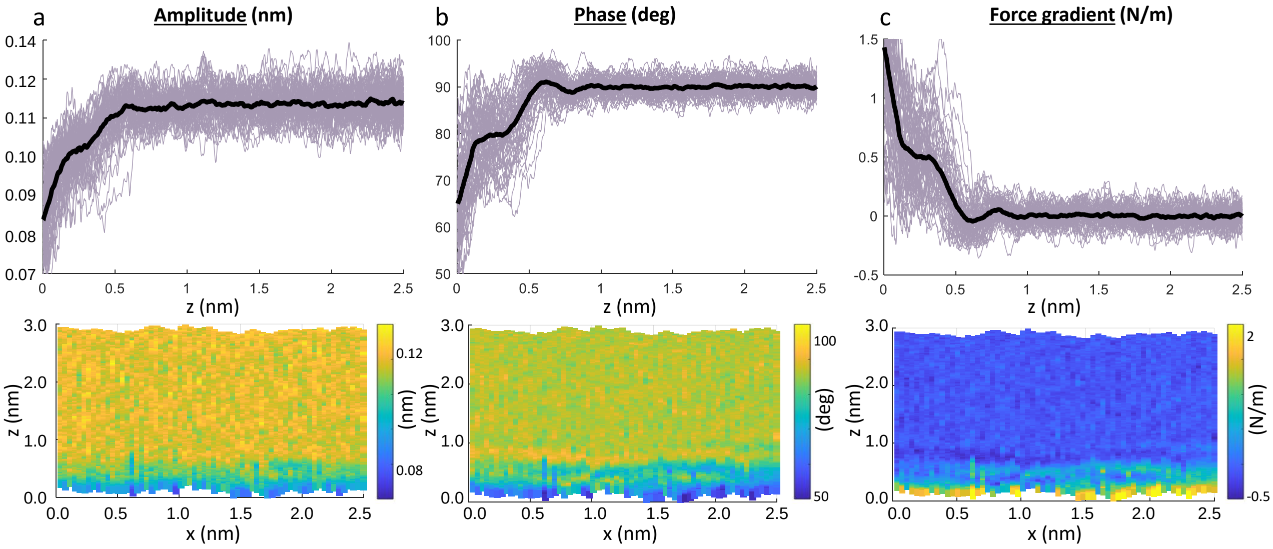

Figure S1: Amplitude (a), phase (b) and reconstructed force gradient (c) vs tip-sample distance profiles corresponding to xy panels (shown below) obtained at the interface of DMPC SLBs in DI water. The average curve is plotted as a black thick line.

S2 3D AFM on S_o and L_d lipid phase

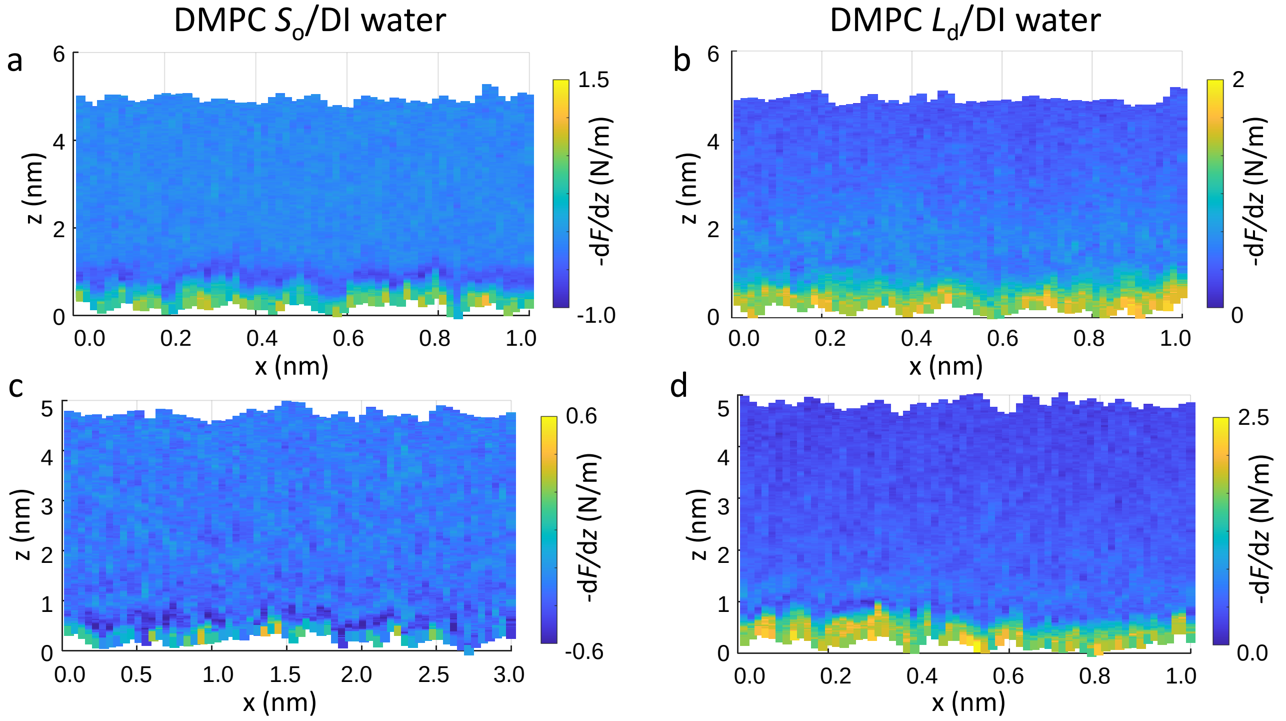

Figure S2: Force gradient xy panels obtained at the interface of DMPC SLBs in the S_o phase (a,c) and L_d phase (b,d) in DI water. These are the corresponding xy panels of the force gradient vs distance curves shown in Figure 4 of the main text.

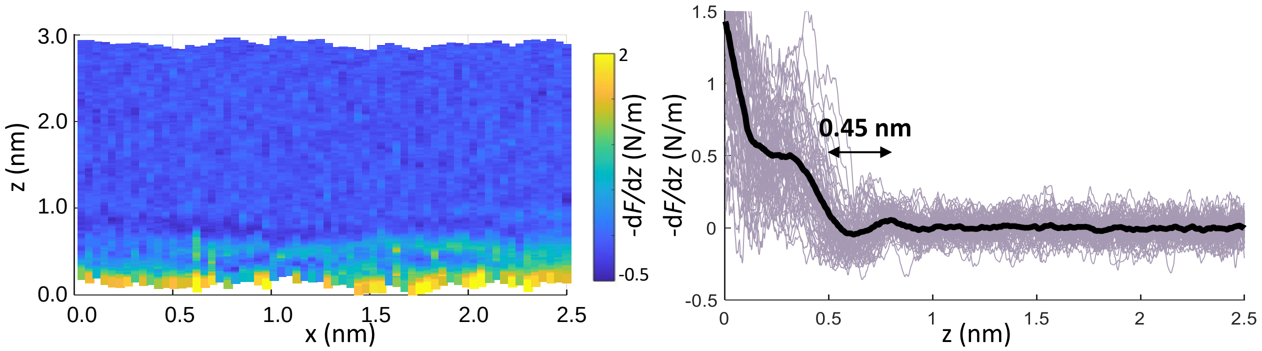

Figure S3: Additional force gradient xy panel obtained at the interface of S_o phase DMPC SLBs in DI water, together with its corresponding force gradient vs distance curve. The interface is characterised by two layers with an interlayer distance of 0.45nm.

S3 Force independence of the salt concentration

To discard the effect of the ionic concentration on the measured force curves, additional experiments on L_d phase lipid membranes were performed in DI water and immediately after in 100 mM KCl solutions. The same cantilever was used to minimise the effect of possible changes in the tip radius. The results are shown in Figure S4. Indeed, we did not see any difference in the force curve trend due to the presence/absence of ions in the solution. This result complements those already shown in the literature for crystalline materials in aqueous solutions. In fact, 3D AFM data obtained using ultrasharp tips never showed a dependent behaviour with respect to the concentration of the salt solution, except for at very high concentrations near saturation.[3,4]

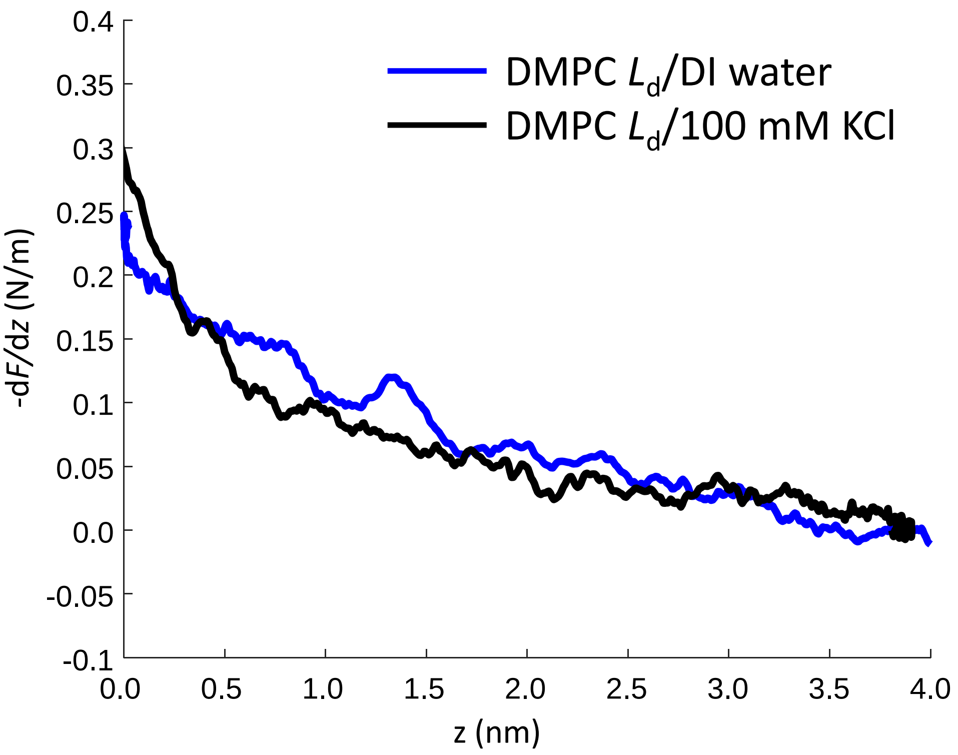

Figure S4: Force gradient vs distance curves at the interface of a L_d phase lipid bilayer with DI water (blue line) and an aqueous solution of 100 mM KCl (black line). The two curves do not show any significant difference.

S4 Fitting procedure for force gradient vs distance curves

The fitting of the data used to extract the monotonic decay of the force gradient is based on the one previously reported by van Lin et al.,[5] where the force-distance dependence is described using a combination of oscillatory and monotonic exponential decay as

$$F(z) = F_o cos(2\pi z/d + \phi)e^{-z/\lambda_o} + F_m e^{-z/\lambda_m} \tag{2}$$

where F_o and F_m are the magnitudes of the oscillatory and monotonic contribution to the force, ϕ is the phase shift, d is the size of the liquid molecule at the interface, and λ_o and λ_m are the decay lengths of the oscillatory and monotonic contribution. Hence, the derivative of this equation gives the function used to fit the experimental data

$$\frac{dF}{dz} = ((2\pi/d)^2 + \lambda_o^{-2})^{1/2} F_o cos(2\pi z/d - \phi - atan(2\pi\lambda_o/d))e^{-z/\lambda_o} + F_m/\lambda_m e^{-z/\lambda_m} \tag{3}$$

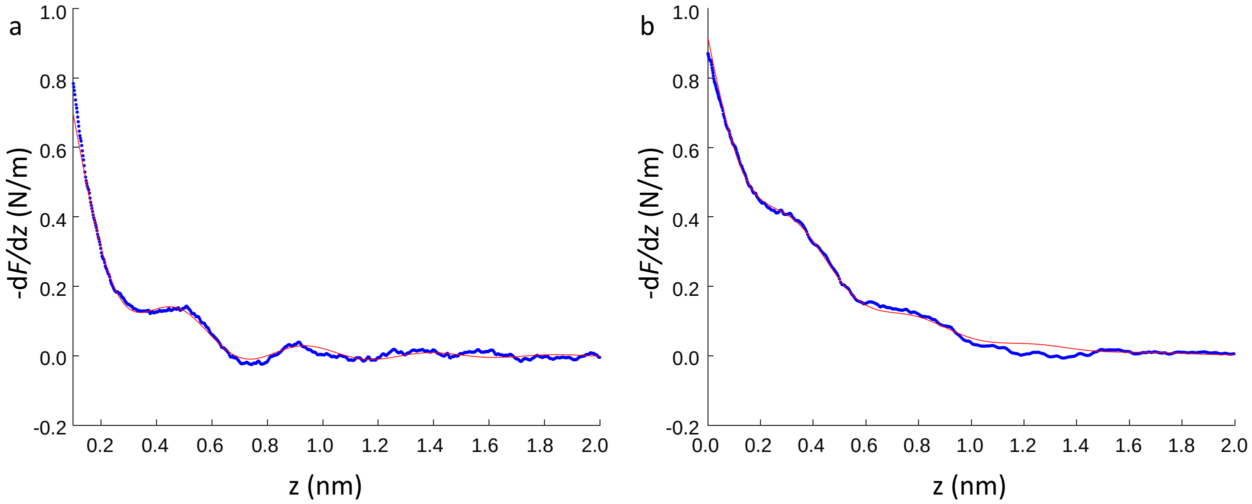

Figure S5: Example of fitting results obtained using Eq. 3 to fit the force gradient vs distance curves at the interface of a S_o (a) and L_d (b) phase lipid bilayer. The red curve is the fitting to the experimental data (blue curve).

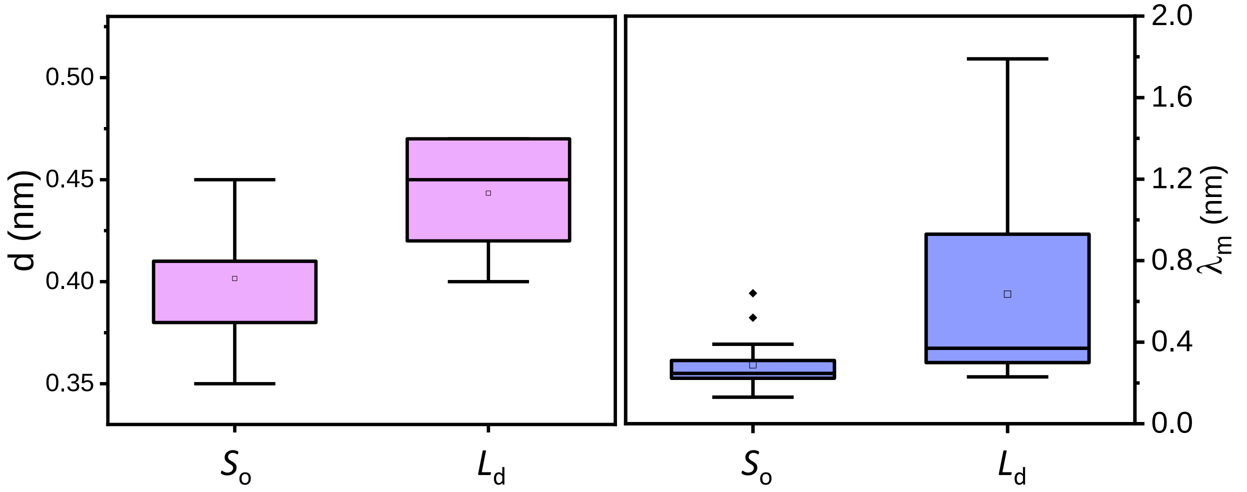

Figure S6: Box plots representing the distribution of the interlayer distances d (in purple) and the decay length of the monotonic component of the force λ_m (in blue) for the 3D AFM experiments obtained on the S_o and L_d phases.

References

1 S. de Beer, D. van den Ende and F. Mugele, *Nanotechnology*, 2010, **21**, 325703.

2 H. Söngen, R. Bechstein and A. Kühnle, *Journal of Physics: Condensed Matter*, 2017, **29**, 274001.

3 S. Benaglia, M. R. Uhlig, J. Hernández-Muñoz, E. Chacón, P. Tarazona and R. Garcia, *Physical Review Letters*, 2021, **127**, 196101.

4 I. Siretanu, S. van Lin and F. Mugele, *Faraday Discussions*, 2023, **34**, 240–241.

5 S. R. Van Lin, K. K. Grotz, I. Siretanu, N. Schwierz and F. Mugele, *Langmuir*, 2019, **35**, 5737–5745.
